# Revisiting the Global Epidemiology of Cholera in Conjunction With the Genomics of *Vibrio cholerae*

**DOI:** 10.3389/fpubh.2019.00203

**Published:** 2019-07-23

**Authors:** Thandavarayan Ramamurthy, Ankur Mutreja, François-Xavier Weill, Bhabatosh Das, Amit Ghosh, Gopinath Balakrish Nair

**Affiliations:** ^1^Centre for Human Microbial Ecology, Translational Health Science and Technology Institute, Faridabad, India; ^2^Department of Medicine, Addenbrooke's Hospital, University of Cambridge, Cambridge, United Kingdom; ^3^Unité des Bactéries, Pathogènes Entériques, Institut Pasteur, Paris, France; ^4^Department of Bacteriology, National Institute of Cholera and Enteric Diseases, Kolkata, India; ^5^Rajiv Gandhi Centre for Biotechnology, Trivandrum, India

**Keywords:** *Vibrio cholerae*, seventh cholera pandemic, single nucleotide polymorphism, whole-genome sequence, CTX phage, CT-genotype

## Abstract

Toxigenic *Vibrio cholerae* is responsible for 1.4 to 4.3 million cases with about 21,000–143,000 deaths per year. Dominance of O1 and O139 serogroups, classical and El tor biotypes, alterations in CTX phages and the pathogenicity Islands are some of the major features of *V. cholerae* isolates that are responsible for cholera epidemics. Whole-genome sequencing (WGS) based analyses of single-nucleotide polymorphisms (SNPs) and other infrequent genetic variants provide a robust phylogenetic framework. Recent studies on the global transmission of pandemic *V. cholerae* O1 strains have shown the existence of eight different phyletic lineages. In these, the classical and El Tor biotype strains were separated as two distinctly evolved lineages. The frequency of SNP accumulation and the temporal and geographical distribution supports the perception that the seventh cholera pandemic (7CP) has spread from the Bay of Bengal region in three independent but overlapping waves. The 2010 Haitian outbreak shared a common ancestor with South-Asian wave-3 strains. In West Africa and East/Southern Africa, cholera epidemics are caused by single expanded lineage, which has been introduced several times since 1970. The Latin American epidemics that occurred in 1991 and 2010 were the result of introductions of two 7CP sublineages. Sublineages representing wave-3 have caused huge outbreaks in Haiti and Yemen. The Ogawa-Inaba serotype switchover in several cholera epidemics are believed to be due to the involvement of certain selection mechanism(s) rather than due to random events. *V. cholerae* O139 serogroup is phylogenetically related to the 7CP El Tor, and almost all these isolates belonged to the multilocus sequence type-69. Additional phenotypic and genotypic information have been generated to understand the pathogenicity of classical and El Tor vibrios. Presence of integrative conjugative elements (ICE) with antibiotic resistance gene cassettes, clustered regularly interspaced short palindromic repeats-associated protein system and *ctxAB* promoter based ToxRS expression of cholera toxin (CT) separates classical and El Tor biotypes. With the availability of WGS information, several important applications including, molecular typing, antimicrobial resistance, new diagnostics, and vaccination strategies could be generated.

## Introduction

Cholera is caused by pathogenic strains of *Vibrio cholerae* due to their colonization in the intestinal milieu and secretion of cholera toxin (CT). The clinical consequences of this diarrheal disease include discharge of substantial volumes of watery stool, loss of electrolyte, rapid dehydration that may advance to hypovolemic shock and metabolic acidosis. Death rates due to cholera infection are reported to be as high as 70%, mainly due to the delay in rehydrating the patients. The global burden of cholera is estimated to be between 1.4 and 4.3 million cases with about 21,000–143,000 deaths per year (1). In 2017 alone, 34 countries reported a total of 1,227,391 cases and 5,654 deaths (2).

Although there are more than 200 serogroups of *V. cholerae*, epidemics of cholera are caused by two serogroups i.e., O1 and O139. The serogroup O1 is classified into two biotypes, classical and El Tor and each biotype into Ogawa and Inaba serotypes. This disease has marked 200 years, with the first cholera pandemic documented in 1817. The first six pandemics were caused by the classical biotype of *V. cholerae* serogroup O1. The ongoing seventh cholera pandemic (7CP) is caused by the El Tor biotype, which appeared in Indonesia in 1961 and reached South Asia after 2 years. In the succeeding years, the El Tor vibrios reached Africa (1970s), South-America (1990s), and the Caribbean Islands (2010) (3). In 1992, serogroup O139 emerged in the Indian subcontinent and spread across Asia until mid-2000s (4). However, this serogroup was eventually superseded by O1, which continues to cause cholera today.

Even though the El Tor vibrios are genetically homogenous, WGS (whole-genome sequence) analysis has identified several lineages (L)/transmission types (T). The newly identified El Tor hybrids, variation in the B subunit of the CT encoding gene CT-B subunit (*ctxB*), polymyxin-B sensitive El Tor vibrios have complicated the biotype differentiation. Recent endemic and epidemic cholera in Asia and Africa are increasingly being attributed to atypical El Tor variants with genetically variable CTX phages, *rstR* genotypes and single-nucleotide polymorphisms (SNPs) throughout the genome of *V. cholerae* (5–7).

The conventional methods such as serotyping, phage typing, antibiogram etc., provide information to distinguish *V. cholerae* isolates involved in outbreaks, but they are inadequate for the clonal definition. In the pre-genomic era, application and interpretation of the results produced using various molecular typing methods had no standardized clonal description and had low discrimination for phylogenetic inference. In order to overcome these complications, WGS based phylogenetic analysis and comparative genome analysis have been used to study the population structure and evolution of *V. cholerae* as well as their epidemiological significance. Herein, we aim to review the outcomes of the recent genomics of *V. cholerae* focusing on the global epidemiology of cholera and subtle genetic changes.

## Features Considered in The Evolution of *V. Cholerae*

Genomic comparison of *V. cholerae* isolates is able to provide critical evidence in understanding the emergence and spread of epidemic cholera. In this, the Bayesian approach (3, 8) supports to time the important nodes with strong temporal signal. The population structure on the other hand might be easily studied by a maximum likelihood approach. WGS-based Bayesian phylogeographic analysis is now being widely used to detect the prototypes of spread, derivation time of species, diversification rates and its time estimation. Using this model, several thousands of WGS of *V. cholerae* O1 genomes have been analyzed and several lineages identified.

*V. cholerae* O1 and O139 isolates were originally categorized in 8 different phyletic lineages (L1 to L8). The classical and El Tor clades are placed in distinct lineages, L1 and L2, respectively and separated by 20,000 SNPs (3). The lineage L3 represents USA Gulf Coast isolates; L5, L6, and L8 are with typical El Tor/O139 vibrios and isolates of L4 and L7 lineages have distinct genetic backbones (3). Six different stages in the molecular evolution of the *V. cholerae* El Tor biotype, from the earliest available isolates (1930s) to the pandemic strain, have also been described (9). The 7CP first emerged in Indonesia and repeatedly spread from the Bay of Bengal to different parts of the world in the form of three waves as determined by the maximum-likelihood phylogenetic tree based on SNP differences across the core genome of *V. cholerae* (3). It took almost 50 years for the pathogen to adapt itself from wave-1 to wave-3 strains of today.

**Figure 1:**
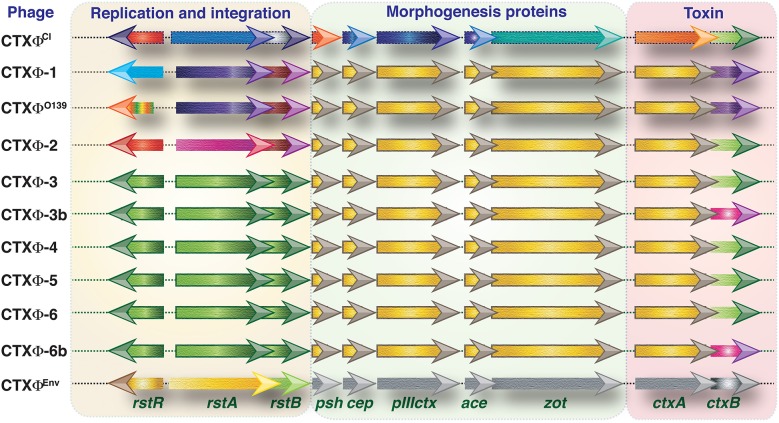
Genetic structure of CTX phages. Different classes of CTXϕs present in the genome of toxigenic *V. cholerae* strains. Except CTXϕ^Env^ all other phages are from the clinical isolates of *V. cholerae*. Types of CTXϕ are classified mainly based on their origin of isolation. Functions associated with replication and integration, morphogenesis and toxin production are clustered into three segments. Arrows indicate different open reading frames (not shown to scale). Directions of arrows indicate direction of transcriptions. Similar color indicates identical DNA sequences. The difference in color indicates variances in DNA sequences.

Based on the amino acid positioning in the CT-B of *V*. *cholerae*, several CT genotypes have been reported (7) (Table 1). The CT phage (CTX) is a lysogenic filamentous phage that harbors the CT encoding genes (*ctxAB*). In the evolution of *V. cholerae*, the CTX has undergone several changes, mainly due to recombination between two major types of CTX (CTX^cla^ and CTX-1) and a satellite phage (RS1) along with point mutations in *ctxB*. Different components and types of CTX phages are shown in Figure 1. RS1 has genes that permit phage DNA replication (*rstA*), integration (*rstB*), regulation (*rstR*), and *rstC*, whose function is unspecified (10, 11). CTX phages are classified mostly by *rstR* and *ctxB* genotype and further subdivided by SNPs throughout the *V. cholerae* genome. CTX^cla^ and CTX^USGulf^ has the classical type of *rstR* and CT genotype-1 (*ctxB1*). CTX^AUS^ also has a classical type of *rstR* but with CT genotype-2 (*ctxB2*). The recorded genetic differences in the *rstA* and *rstB* of various CTX in 7CP are summarized in Table 2. The type of lineage, CT genotype and CTX phage type are collectively used in defining the wave pattern or clade. The CTX phage arrays (TLC-truncated CTX-CTXΦ^*B*3^, or TLC-CTXΦ^*B*3^-CTXθ^*B*3^-RS1), specifically detected in Latin American isolates were not reported in El Tor, altered El Tor, or El Tor variants from Asia, Africa, and Haiti (12).

**Table 1:** CT-genotypes of *V. cholerae* O1/O139.

**CT-genotype (*ctxB*)**	**Nucleotide position**									**Amino acid position**								
	**58**	**72**	**83**	**101**	**106**	**115**	**138**	**165**	**203**	**20**	**24**	**28**	**34**	**36**	**39**	**46**	**55**	**68**
1	C	A	A	A	A	C	T	A	C	H	Q	D	H	T	H	F	K	T
2	C	A	A	A	A	C	G	A	C	H	Q	D	H	T	H	L	K	T
3	C	A	A	A	A	T	T	A	T	H	Q	D	H	T	T	F	K	I
4	C	A	A	A	A	T	T	A	C	H	Q	D	H	T	Y	F	K	T
5	C	A	C	A	A	C	T	A	C	H	Q	A	H	T	H	F	K	T
6	C	A	A	C	A	T	T	A	C	H	Q	D	P	T	Y	F	K	T
7	A	A	A	A	A	C	T	A	C	N	Q	D	H	T	H	F	K	T
8	C	C	C	A	A	C	T	A	C	H	H	A	H	T	H	F	K	T
9	C	A	A	A	A	C	G	C	C	H	Q	D	H	T	H	L	N	T
10	C	A	A	C	A	T	T	A	T	H	Q	D	P	T	Y	F	K	I
11	C	A	A	C	A	C	T	A	C	H	Q	D	P	T	H	F	K	T
12	C	A	A	A	G	T	G	C	C	H	Q	D	H	A	Y	L	N	T

**Table 2:** Differences in the sequence of *rstA* and *rstB* in various types of CTX phages detected in 7PC *V. cholerae*.

**CTX type**	***rstR* type**	***ctxB***	***rstA*** **sequence**							***rstB*** **sequence**										
			**27**	**162**	**183**	**258**	**927**	**933**	**942**	**74-76**	**87**	**93**	**105**	**189**	**360**	**364**	**366-368**	**371-372**	**379**	**381**
CTX-1	El Tor	3	C	C	C	G	T	C	G	GTA	A	T	G	A	A	C	ACC	TT	T	A
CTX-3	El Tor	1	C	C	C	G	C	T	T	GTA	A	T	G	A	A	C	ACC	TT	T	A
CTX-3b	El Tor	7	C	C	C	G	C	T	T	GTA	A	T	G	A	A	C	ACC	TT	T	A
CTX-4	El Tor	1	C	C	C	G	C	T	T	Δ	A	T	G	A	A	C	ACC	TT	T	A
CTX-5	El Tor	1	C	C	C	G	C	T	T	Δ	T	C	A	A	A	C	ACC	TT	T	A
CTX-6	El Tor	1	C	C	C	G	C	T	T	Δ	T	C	A	G	A	C	ACC	TT	T	A
CTX-6b	El Tor	7	C	C	C	G	C	T	T	Δ	T	C	A	G	A	C	ACC	TT	T	A
CTX-2	Class	1	T	T	A	C	T	C	G	Δ	T	C	G	A	A	C	ACC	TT	T	A

Δ, *deletion of nucleotides. Similar color indicate identical nucleotides in each CTX type*.

Horizontal gene transfer (HGT) plays a major role in microbial evolution, allowing microbes to acquire new genes and phenotypes. *V. cholerae* O139 serogroup might have evolved genetically from *V. cholerae* serogroup O1 through the alteration of its somatic (O) antigen structure by means of mutations in the O antigen region of El Tor strain or acquisition of El Tor virulence genes by an unknown non-O1 (e. g., O22) *V. cholerae* (13, 14). SNPs based phylogenomic tree has placed the several O139 isolates into a close cluster, indicating their discrete derivation from the O1 serogroup (15). Despite the presence of two CTX types, (CTX-2 and CTX-3) the O139 isolates descent into one cluster in the wave-2 (3, 16).

Other than the core genome, evaluation of insertion or deletion of bases (indels) and gene elements by HGT allow more resolution to differentiate genetically related isolates. This is because *V. cholerae* is naturally capable of internalizing the DNA from the environment (17). HGT and recombination events made a large impact on the transmission of virulence factors, antimicrobial resistance genes and on serogroup/serotype modifications in *V. cholerae* (serogroup O1 to O139; serotype Ogawa with AB antigen to Inaba with AC antigen). A rare Hikojima serotype has also been described that expresses all three antigens (A, B, and C). Evidence indicates that the Hikojima serotype is a transient serotype when *V. cholerae* O1 undergoes serotype switching from Ogawa to Inaba (18).

Two genomic regions of the *Vibrio seventh* pandemic island, VSP-I and VSP-II were found to be unique to the El Tor and O139 vibrios (19, 20). In El Tor vibrios, VSP-II is a part of a novel genomic island (GEI) encompassing VC0490-VC0516. Several variants in this VSP-II have been reported from many countries (12, 21–24). The wave-1 West African–South American (WASA) clade is differentiated from all other *V. cholerae* by gaining novel VSP-II genes (20) and a genomic island (WASA-1) (3). VSP-II gene variants (deletions of VC0495-VC0498, VC0495-VC0500, or VC0495-VC0512) were found in wave 3 7CP isolates. These deletions might have been driven by IS*Vch4*, which was inserted in VC0495 in early wave-3 isolates (Table 3). WASA-1 genomic island was also identified in African and Latin American isolates during the late 1980s (T1, LAT-1). *V. cholerae* O1 isolated between 2007 and 2014 from Kolkata were divided into lineage 1, which had VSP-IIB and *ctxB1*, and lineage 2 that contained VSP-IIC and *ctxB7* (24).

**Table 3:** Inferred origin and transmission events of *V. cholerae* O1 with cholera waves, phylogenetic sublineages of introduction events (T), and Latin American transmission (LAT).

**Wave/sub-lineage**	**MRCA in years**	**Spread from**	**Spread into**	**Features**	**References**
Wave-1	1938–61	Indonesia	Indian S	c*txB3*, CTX-1 with *rstR*^ElTor^ on chrom-1, no ICE SXT/R391	(3)
	1967–71	Indian SC	South-East Asia		
	1975–85	Indian SC	East Asia		
	1967–89	Indian SC	Mozambique		
	1969–81	Indian SC	Angola		
	1973–78	Indian SC	Middle East		
	1974–75	Indian SC	East-Europe		
	1973–85	Indian SC	Ethiopia		
	1981–85	Angola	US-Gulf coast Latin America		
Wave-2	1990–93	Indian SC	South-East Asia	c*txB1* and tandem repeat of CTX-2 on chrom-2, c*txB4 to* c*txB6* in O139, ICE SXT/R391	(3)
	1992–02	South-East Asia	East Africa		
Wave-3	1989–97	Indian SC	Nairobi Tanzania Djibou	c*txB1* or c*txB7 rstR*^ElTor^ in CTX3 to CTX6, ICE*Vch*Ind5	(3)
	2003–07	Indian SC	South-East Asia		
	2005–09	Indian SC	Haiti		
T1 (wave-1)	1970–75	Middle East	North Africa, West Africa, East Africa	c*txB3*, VSP-II var-WASA, WASA-1	(31)
T2 (wave-1)	1989–91	West Africa	Latin America	c*txB3*, VSP-II, WASA-1	
T3 (wave-1)	1970	Middle East	East Africa	c*txB3*, VSP-II	
T4 (wave-1)	1970–78	Middle East	East Africa	c*txB3*, VSP-II	
T5 (wave-1)	1970–72	Middle East	East Africa, Central Africa	c*txB3*, VSP-II, IncA/C plasmid	
T6 (wave-1)	1986–89	Middle East	East Africa	c*txB3*, VSP-II	
T7 (wave-2)	1982–84	East Asia	North Africa, West Africa	*ctxB3*, VSP-II var-Cameroon,	
T8 (wave-2)	1994–98	Middle East	East Africa, South Africa	c*txB1*, ICE*Vch*Ban9	
T9 (wave-3)	1988–91	South-East Asia	West Africa	c*txB1*, VSP-II (VC0495::ISVch4), ICE*Vch*Ind5	
T10 (wave-3)	1991–95	South-East Asia	East Africa, Central Africa	*ctxB1*, VSP-II (ΔVC0495-VC0498), ICE*Vch*Ind5	
T11 (wave-3)	2001	South-East Asia	East Africa, South Africa	c*txB1*, VSP-II (ΔVC0495–VC0512), ICE*Vch*Ind5	
T12 (wave-3)	2007	South-East Asia	West Africa	c*txB7*, (ΔVC0495–VC0512), ICE*Vch*Ind5	
T13 (wave-3)	2015–16	Indian SC	East Africa and Yemen	c*txB7, tcpA*^CIRS101^ VSP-II (ΔVC0495–VC0512), ICE*Vch*Ind5/Ban5	(31)
LAT-1 (wave-1)	1989	Western and Central Africa	Latin America	c*txB3*, VSP-II var-WASA, WASA-1	(7)
LAT-2 (wave-1)	1987–1989	South/South-East Asia	Central America	c*txB1* in Chrom-2	
LAT-3 (wave-3)	2010	South Asia	Haiti, Latin America	*ctxB7*, VSP-II (ΔVC0495-VC0512), ICE*Vch*Ind5/Ban5	

## Present Status of Classical *V. Cholerae* O1

Globally, the 7CP is dominated by the El Tor vibrios. Existence of *V. cholerae* O1 classical biotype was reported from Bangladesh during 1961–1968 and 1982–1992 (25), Mexico during 1991–1997 (26) and Thailand in 2000 (27). This indicates that the classical biotype actually persisted longer than previously thought. The Bangladesh classical vibrios isolated in two events remained clonally distinct (25). WGS based phylogenetic analysis displayed that classical biotype isolates in Mexico are part of the classical lineage, and not derived from the Gulf Coast lineage (8).

## Cholera Waves Triggered by El Tor Vibrios

Based on several molecular features, *V. cholerae* has been classified into three waves of cholera (3). The features of each wave are as follows:

### Wave-1

The wave-1 spanned 1961–1999. Isolates belonging to this wave contains CTX-1 that harbors *rstR*^ElTor^ on chromosome 1, CT genotype-3 (*ctxB3*), but not the ICE of the SXT/R391 family. *V. cholerae* O1 of this wave may have ascended with the addition of TLC element and then by CTX-1 and RS1 element. Wave-1 spread from Indonesia to the Bay of Bengal region in the Indian subcontinent and then to East Africa and South-West Americas (Table 3).

### Wave-2

Wave-2 isolates were dominant between 1978 and 1984. Isolates of this wave were first identified in the Indian subcontinent. These isolates have *ctxB1* and tandem repeat of CTX-2 on chromosome 2. Some of them may contain CTX-1 and/or RS1 on chromosome 1. Wave-2 spread to East Asia and Africa (Table 3). CT genotypes-4 to 6 (*ctxB4-ctB6*) were detected in *V. cholerae* O139 isolated from Bangladesh during 1999–2005 (28). Isolates of this wave may or may not contain R391 family ICE-SXT element (3).

### Wave-3

The wave-3 isolates harbor CT-genotype 1 (1991–2010) or CT-genotype 7 (2010~). Wave-3 isolates mainly have *rstR*^ElTor^:TLC:RS1:CTX3 to CTX6. Based on the genetic differences, these isolates are subcategorized into three or more sub-clades. The wave-3 clade with *ctxB7* allele has emerged from Kolkata, India in 2006 (29) and continues to spread globally, as seen in the Haiti, Yemen and other recent cholera epidemics. *V. cholerae* isolated from a patient during an outbreak of cholera in Mariupol, Ukraine, in 2011 also contained the *ctxB7* (30). Almost all the *V. cholerae* isolates belonging to wave-3 contain ICE of the SXT/R391 family, mostly the ICE*Vch*Ind5.

## Global Transmission of Cholera

Historically, cholera has been considered to have originated in Asia and spread from there. All the three waves began with Asiatic cholera. China has played an important role in the earliest global transmission of 7CP after its appearance in Indonesia (31). *V. cholerae* O1 had entered China four times from South Asia (1975–2004). From China, cholera transmission has occurred in South Asia (three times during 1967–1999), Indonesia (1960) and Southeast Asia (2007) (31). Based on the phylogenetic analysis, *V. cholerae* representing the cholera wave-3 have been subdivided into several clades (Table 3). This includes T1–T12 from African countries (32) LAT-1 to LAT-3 from Latin Americas (7) and T13 from East Africa and Yemen (33). Several monophyletic clades in *V. cholerae* O1 isolated in China (31), in these, clade 3.A links African epidemic isolates that may be imported from South Asia, clades 3.B contains isolates representing Haiti outbreak as well as from South Asia. In China, cholera outbreaks in 2008 and 2010 were caused by isolates that represented clade 3.B from South Asia (31).

The causative pathogen of the Haiti 2010 outbreak was from a Nepal cluster in 3.C, which is consistent with previous reports (34, 35). An isolate from Cameroon in 2010 was assumed to be a close genetic relative of the Haiti outbreak strain (31). However, phylogeny based on the SNPs within the orthologous core genes revealed a closer relationship between Haitian and Indian isolates than with those from Cameroon (36). Cholera outbreaks have been reported in Russia during 1990–2001, mostly from Stavropol, Republic of Dagestan, Vladivostok, and Yuzhno-Sakhalinsk. *V. cholerae* O1 El Tor isolates from Siberia and the Far East during 1990s belonged to wave-2 and-3 with CT-genotype 1 (37). A new CTX prophage variant (CTX-2a) linking the traits of CTX-2 and CTX-1 was identified. Phylogenetic analysis distinguished the common ancestors of Russian isolates from Bangladesh and Turkey (37).

During 2016–2018, Yemen faced the largest documented cholera epidemic with 1,103,683 cases and 2,385 deaths (38). Yemen cholera epidemic originated from a recently emerged 7CP wave-3 clade, which contains the *ctxB7*. This clade has been identified as sublineage T12 in West Africa in 2008, Haiti in 2010 and as T13 in East Africa between 2013 and 2014 before its spread in Yemen (33). Phylogenetic analysis revealed that the *V. cholerae* O1 isolates from Yemen are different from those in circulation in the Middle East countries where cholera was previously identified. The Yemen isolates are similar to isolates (T13) found in the Eastern African countries, such as Kenya, Tanzania and Uganda in 2015–2016 (33). *V. cholerae* that belongs to sub-lineage T13 includes the presence of a variant *tcpA* (*tcpA*^CIRS101^) and a deletion within VSP-II (VC0495–VC0512) (33).

### Cholera in the Americas

Cholera was not reported from Latin America for nearly 100 years. Cholera cases representing small outbreaks have been reported in 1970s from the Gulf Coast (Louisiana and Texas) of the United States and in Mexico during early 1980s (39). The Latin American cholera epidemic began in January 1991 along the Pacific coast of Peru. In the subsequent 6 years, cholera spread swiftly to Ecuador, Colombia, Chile, Brazil, the US, Mexico, Guatemala, Bolivia, and El Salvador, infecting 1.2 million people with 12,000 deaths until 1997 (40). WGS-based maximum likelihood phylogeny analysis identified the epidemic clone as Latin American transmission (LAT-1) sublineage containing *ctxB3*. This sublineage corresponded to the T2 sublineage described by Weill et al. (33). Western Africa experienced cholera outbreaks caused by T2 during the 1980s and hence this region would have been the source for the LAT-1/T2 sublineage. This sublineage was still found during 2004–2010 in Mexico and it harbored a truncated CTXφ duplication, which is unique among the 7CP strains (41).

The introduction of the LAT-2 sublineage to Mexico might be from South Asia or China or introduction via Eastern Europe. Introduction of wave-2 second sub-lineage (LAT-2) may have occurred during 1987–1989, parallel with that of the LAT-1 introduction. In 2010, the 7CP was introduced into Haiti, affecting about 800,000 with 9,600 deaths (42). The Haitian isolates of *V. cholerae* O1 were distinguished as LAT-3, has its origin from the Asia region. Even though *V. cholerae* O1 biotype El Tor was identified in most of the cholera cases, the classical biotype was identified in Mexico until 1997 (26, 41). Previous investigations identified 11 different lineages in Latin American *V. cholerae* O1 isolates. Some of these include, the Gulf Coast lineage (43), ribotypes MX1 to MX3 lineages in Mexico (44), endemic Latin American 1 (ELA-1) lineage from Brazil and Argentina (45, 46). These results display that the Latin American cholera epidemics were due to multiple introductions and not derived from indigenous local lineages. In addition, *ctxB3, ctxB1*, and *ctxB7* were identified in LAT-1, LAT-2, and LAT-3, respectively.

### African Cholera

The 7CP first emerged during 1970, in the Middle East and West Africa with more than 150,000 cases and 20,000 deaths (47, 48). After these initial outbreaks, cholera became endemic in many African countries. During 1970–2011, Democratic Republic of the Congo, Mozambique, Nigeria and United Republic of Tanzania were affected by large number of cholera cases (260,000–390,000) and deaths (11,000–25,000) (49). Previous molecular studies could not give substantial evidence about the origin and spread of *V. cholerae* from these outbreaks. The genomic approach has identified several transmission events of cholera from South Asia to Africa (T1, T3–T13) and from Africa to America (T2) or to Asia (T13) (32). Of these, T1–T5, T6–T8, T9–12 belongs to wave-1, wave-2, and wave-3, respectively (32). T1 occurred during the 1970s in South/East Asia and trailed to Middle East and Russia.

In Europe, only imported cases of cholera have been reported in the recent past. The European T1 isolates of early 1970s had its origin in West or North Africa. T2 (1989–1991) was responsible for the spread of cholera from West Africa to Peru for the Latin American epidemic during 1990s (Table 3). In Northeastern Africa, T3 (1970), T4 (1970–1978) and T5 (1970–1972) were associated with several outbreaks originating from the Middle East. T5 was linked with large outbreaks in Rwanda in 1994. The T6 sublineage was also imported from the Middle East to the Horn of Africa/East Africa during 1986–1989. The South Asia origin T7 (1982–1984) was detected in isolates from several outbreaks in North and West Africa. T8 sublineage from the Middle East was associated with outbreaks in South Africa in 2001–2002. Zimbabwe outbreaks in 2008–2009 were linked with T8 and T11. Most of the African countries were affected by T9–T12 during 1990–2014 and these sub-lineages originated in South Asia.

## Alterations in Antimicrobial Resistance

As an adjunct to rehydration therapy, antibiotics have been used in the treatment of cholera to shorten the duration of diarrhea and to limit the bacterial spread. Over the years, resistance to many useful antibiotics developed, which include chloramphenicol, furazolidone, trimethoprim-sulfamethoxazole, nalidixic acid, tetracycline, and fluoroquinolones (50). Antibiotic resistance profiles have also been used to designate the *V. cholerae* strains in the epidemiology of cholera. Since the 1960s, many resistance phenotypes of *V. cholerae* have been reported in Asian regions. In Africa, the first antibiotic-resistant *V. cholerae* isolates was described in Tanzania during 1979 (32). The genetic basis of antibiotic resistance is mostly due to the existence of genes conferring drug resistance in the plasmids, chromosomal mutations and ICEs of the SXT/R391 family. Incompatibility (Inc) group A/C plasmids are stably maintained in *V. cholerae* that emerged during the 7CP (51). Some isolates of T1, T5, T6, and T10 *V. cholerae* from West/East Africa isolates were shown to contain a multidrug resistance encoding IncA/C plasmid (32).

ICEs are mobile genetic elements integrated into the bacterial genome. In *V. cholerae*, the R391 family ICEs play a crucial role in acquiring antimicrobial resistance genes and are widely used as one of the markers of clonal expansion of the pathogen. The ICE-SXT element was first reported in *V. cholerae* serogroup O139 from India that emerged during 1993 (52). Based on the WGS phylogenetic analysis of wave-2 7CP isolates, the calculated acquisition time of SXT element was between 1978 and 1984, much earlier to its detection in the O139 serogroup (53). This interpretation corresponds with the discovery of SXT in a *V. cholerae* O1 isolate from Vietnam during 1990 that was devoid of any resistance gene cassettes (54). Resistance to 2,4-diamino-6, 7-diisopropylpteridine (vibriostatic agent O/129) has been strongly correlated with chloramphenicol, streptomycin, trimethoprim-sulfamethoxazole resistance in *V. cholerae* O1 isolated during 1989–1990 in Kolkata, India (55). However, the role of ICE-SXT/R391 was not determined in these isolates.

SXT/R391 ICE has been detected in all most all the isolates that represented wave-2 and wave-3 (3). SXTs are reported to display polymorphism, with their genetic backbone and resistance encoding genes to chloramphenicol (*floR*), streptomycin (*strA* and *strB*), trimethoprim (*dfrA18* and *dfrA1*), sulfamethoxazole (*sul2*), and tetracycline (*tetA* and *tetR*). The type of SXT ICE element is specific to geographical regions. More than 30 ICEs have been grouped within the SXT/R391 family of clinical and environmental *V. cholerae* isolates (56). Worldwide, ICE*Vch*Ind5/ICE*Vch*Ban5 is very common among *V. cholerae*. In Africa, sublineages T9 to T13 that originated from South Asia carried SXT/R391 with ICE*Vch*Ind5/ICE*Vch*Ban5 (32, 33). SXT/R391 ICE was absent in the epidemic *V. cholerae* O1 isolated in Latin America in the 1990s (3, 12). Deletion of ICE*Vch*Ind5/ICE*Vch*Ban5-like SXT/R391-ICE including the variable region-III that encodes resistance to streptomycin (*strA* and *strB*), chloramphenicol (*floR)*, and sulfonamides (*sul2)* has also been found in the recent Yemen isolates (33). This trend has been reported in several other 7CP wave-3 isolates (32, 57).

Conventionally, polymyxin-B susceptibility testing is used to differentiate classical and El Tor biotypes, in which the latter is always resistant. Polymyxin-B susceptible *V. cholerae* O1 El Tor isolates emerged in 2012 Kolkata and spread to other parts of India (58, 59), East Africa and Yemen in 2016 (33). A non-synonymous SNV in *vprA* (*VC1320*) i.e., change in the VprA (D89N) was found for this polymyxin susceptible conversion among El Tor *V. cholerae* isolates (33). It is possible to determine the AMR encoding genes using the WGS. However, adequate evidence to support clinical decision-making based on the genome based antibiotic susceptibility testing is still not available (60).

## The Other Important Attributes of *V. Cholerae*

Most of the 7PC isolates of O1 and O139 serogroups belonged to multilocus sequence type ST69 whereas *V. cholerae* O1 classical variants belonged to ST73 (27, 61, 62). Serotype changing of *V. cholerae* O1 from Ogawa to Inaba and back to Ogawa has been seen in many cholera endemic regions. This change is associated with mutational disruption of the methyltransferase encoded in the serotype-determining *wbeT*, which is part of the O1 somatic antigen. Shift in serotypes of Ogawa and Inaba was previously thought due to unknown selection mechanisms based on modifications of the O1 antigen or in the *wbeT* (63, 64). WGS analysis of African isolates of Inaba serotype showed alterations in the *wbeT* gene, including premature stop codons caused by SNPs or short indels, non-synonymous SNPs, insertion sequences, integrations, deletions and disruption of the start codon (32).

Expression of CT in classical and El Tor vibrios depends on many factors. More than 500 genes (13%) have been found to be differentially expressed in classical and El Tor biotypes. Biofilm formation, chemotaxis, transport of amino acids/peptides and iron encoding genes that support environmental survival are expressed at a higher level in the El Tor biotype, whereas expression of *vieSAB* genes encoding *ctxA* transcription is more in the classical biotype leading to its higher virulence (65). ToxRS contribute in the activation of the *toxT* promoter, which encodes another transcriptional activator and triggers other virulence genes. In classical *V. cholerae* isolates, the ToxRS directly activates *ctxAB* in a ToxT-independent manner in the presence of bile acids (66). This mechanism is not found in El Tor vibrios, as there is a difference in the number of heptad repeats (TTTTGAT) in the upstream regions.

Clustered regularly interspersed short palindromic repeats-associated proteins (CRISPR-Cas) are microbial nuclease systems in the chromosome that are involved in defense against entering nucleic acids/bacteriophages. Bacteria also resist phages by hosting phage-inducible chromosomal islands (PICI) which prevent phage reproduction. In classical biotype, CRISPR-Cas is widely present (67), whereas most of the El Tor vibrios lack this system (68). Many clinical El Tor isolates from Bangladesh were found to encode a phage-inhibitory chromosomal island, known as a PICI-like element (PLE) (67). PLE-mediated inhibition of phage replication is common in *V*. *cholerae* O1 El Tor (69). A CRISPR array consists of short, direct repeats divided by several short sequences called spacers. The spacer sequences of the CRISPR arrays carried 100% sequence homology with different regions of the PLE (67).

## Conclusions

Comparative genomic analysis of *V. cholerae* might give answer to several challenging and historical questions that could only be speculated previously. Based on the available scientific information/evidence, action plans have been set to achieve a 90% reduction in cholera deaths in 20 endemic countries by 2030 (70). WGS will form a critical component in accomplishing the sustained developmental goal (SDG) with several important applications including molecular typing, antimicrobial resistance, new diagnostics and vaccination strategies.

## Author Contributions

TR conceptualized and wrote the initial draft. AM and F-XW contributed critical interpretation of the content. AG, BD, and GN critically appraised and revised the manuscript. All the authors read and approved the final manuscript. AG is J. C. Bose Chair Professor of the National Academy of Sciences, India (NASI).

### Conflict of Interest Statement

The authors declare that the research was conducted in the absence of any commercial or financial relationships that could be construed as a potential conflict of interest.
