# Corrigendum: Revisiting the Global Epidemiology of Cholera in Conjunction With the Genomics of *Vibrio cholerae*

**DOI:** 10.3389/fpubh.2019.00237

**Published:** 2019-08-23

**Authors:** Thandavarayan Ramamurthy, Ankur Mutreja, François-Xavier Weill, Bhabatosh Das, Amit Ghosh, Gopinath Balakrish Nair

**Affiliations:** ^1^Centre for Human Microbial Ecology, Translational Health Science and Technology Institute, Faridabad, India; ^2^Department of Medicine, Addenbrooke's Hospital, University of Cambridge, Cambridge, United Kingdom; ^3^Unité des Bactéries, Pathogènes Entériques, Institut Pasteur, Paris, France; ^4^Department of Bacteriology, National Institute of Cholera and Enteric Diseases, Kolkata, India; ^5^Rajiv Gandhi Centre for Biotechnology, Trivandrum, India

**Keywords:** *Vibrio cholerae*, seventh cholera pandemic, single nucleotide polymorphism, whole-genome sequence, CTX phage, CT-genotype

In the original article, there was an error in the title.

The title has been corrected to: “Revisiting the Global Epidemiology of Cholera in Conjunction With the Genomics of *Vibrio cholerae*.”

The authors apologize for this error and state that this does not change the scientific conclusions of the article in any way. The original article has been updated.

